# Ratio Fluorescence Determination of Tetracycline with Europium(III)-Doped Boron Nitride

**DOI:** 10.3390/s25227056

**Published:** 2025-11-19

**Authors:** Shang-Qing Zhang, Xiao-Yan Sun, Kai-Xin Liu, Ming-Li Chen

**Affiliations:** 1 Research Center for Analytical Sciences, Department of Chemistry, College of Sciences, Northeastern University, Shenyang 110819, China; zhangsq@mail.neu.edu.cn (S.-Q.Z.); sunxiaoyan@stumail.neu.edu.cn (X.-Y.S.); liukaixin@stumail.neu.edu.cn (K.-X.L.); 2 Shenyang Institute for Food and Drug Control, Shenyang 110122, China

**Keywords:** ratio fluorescent probe, antenna effect, visual detection, hydrothermal method

## Abstract

It is important to develop a tetracycline (TC) detection method with a simple synthesis method, high sensitivity, and fast detection speed. Herein, a novel sensor was designed using europium-doped boron nitride (BN-Eu) for evaluation on tetracycline (TC). BN-Eu was synthesized by a simple one-step hydrothermal method. Based on the dual-emission fluorescence signal characteristics of BN-Eu, the content of tetracycline was detected by ratio fluorescence sensing. When the TC concentration increased, the fluorescence emission of BN at 449 nm remained nearly constant, the characteristic emission peak of Eu^3+^ at 618 nm was enhanced due to the antenna effect(AE). The ratiometric fluorescence detection of TC in the range of 0.010–1.0 μmol L^−1^ was achieved with a detection limit of 4.0 nmol L^−1^. In addition, the detection system underwent a color shift from blue to red under an irradiation of 365 nm as the TC concentration increased. Based on this, TC visual detection was achieved. The colorimetric signal versus the concentration of TC in the range from 0 to 50 μmol L^−1^ had a good linear relationship with a detection limit of 1.4 μmol L^−1^. The probe showed good detection performance through the determination of tetracycline content in tetracycline ointment. The prepared BN-Eu probe has fast response, good sensitivity to TC, and has good potential in detecting tetracycline content in complex samples.

## 1. Introduction

Tetracycline (TC), the broad-spectrum antibiotic, has a bactericidal effect against both Gram-positive and Gram-negative bacteria and is often used as a kind of bacteriostat for human therapy and animal husbandry. Therefore, various tetracycline ointments have been developed for antibacterial therapy, which inhibited the growth of peptide chains by specifically binding to bacterial ribosomes to affect the synthesis of bacterial protein. However, excessive intake of TC in the human body will lead to increased drug resistance and decreased immunity of the human body, which is harmful to human health, such as tooth discoloration, liver damage, hearing loss, and skin photosensitization [1,2]. Currently, the standard method for quantifying tetracycline in tetracycline ointment relies primarily on microbial assays. Although this approach provides accurate results, it is time-consuming. Consequently, it is significantly important to develop a detection method that is sensitive, rapid, accurate, and cost-effective.

At present, a variety of TC detection methods have been developed, such as capillary electrophoresis (CE) [3], high-performance liquid chromatography (HPLC) [4], enzyme-linked immunoassay (ELISA) [5], surface enhanced Raman scattering (SERS) [6], and fluorescence (FL) [7]. These methods continue to enrich the development of TC detection. Among them, the fluorescence detection is well-known due to its advantages such as rapid determination, time-saving pretreatment, and high sensitivity [8]. Nowadays, many different fluorescent materials, such as MOF [9,10,11], quantum dots [12,13,14], and nanoclusters [15], have been used for the efficient detection of TC drugs, but the sensitivity and accuracy in the process of sensing TC still need to be improved.

The selectivity and sensitivity of lanthanide doped materials in the determination of tetracycline have been unanimously recognized. Due to low accuracy and weak anti-interference ability of the single-signal probe, it has become a research direction of sensing TC to prepare the dual-signal probe-based suitable carriers including carbon nitride nanosheets [16], Ir(III)-doped silicon nanoparticles [17], palygorskite nanomaterials [18], etc. They all combine with Eu^3+^ to exert the potential of sensing TC through the dual-signal sensing detection. However, in these methods, the synthesis steps of most materials are tedious, and the second addition of Eu^3+^ is the response signal, which affects the accuracy of determination. Therefore, it is crucial to develop a simple, sensitive, and fast TC sensing method.

In this work, europium-doped boron nitride (BN-Eu) was synthesized by a one-step hydrothermal method [19]. Based on its good optical properties, BN-Eu was used as a fluorescent probe to detect TC. Taking the characteristic emission of BN-Eu at 449 nm as the fluorescence reference signal and the characteristic emission at 618 nm as the response signal, a ratio fluorescence sensing platform was constructed. After adding TC, TC coordinated with Eu^3+^ of BN-Eu to form chelate and enhance the fluorescence at 618 nm by the antenna effect [20]. Concurrently, the emission at 449 nm is nearly constant. Under the 365 nm irradiation, the solution changed from blue to red. The smartphone collected data and analyzed the RGB value by color recognition software, and obtained the linear relationship between R/B and TC concentration, thus realizing the visual detection of TC. The prepared BN-Eu probe has a fast response and good sensitivity to TC. By measuring the content of tetracycline in real samples, the sensing system proves the good application potential of the sensing method in actual sample detection.

## 2. Experimental Section

### 2.1. Chemical and Materials

Boric acid (H_3_BO_3_) and urea (CO(NH_2_)_2_) were obtained from Sinopharm Chemical Reagent Co., Ltd. (Shanghai, China). Europium chloride hexahydrate (EuCl_3_·6H_2_O, 99.9%), diethyl ether, hydrochloric acid, oxalic acid, glycine (Gly), leucine (Leu), histidine (His), phenylalanine (Phe), methionine (Met), glutathione (GSH), glucose (Glu), fructose (Fru), ampicillin (AMP), ciprofloxacin (Cip), enrofloxacin (Enr), sulfadiazine (SD), vancomycin (Van), doxycycline (DOX), oxytetracycline (OXY), tetracycline (TC), chromatographic methanol, and chromatographic acetonitrile were purchased from McLean Biochemical Technology Co., Ltd. (Shanghai, China). NaOH, CrCl_3_, CoCl_2_, AlCl_3_, CuCl_2_, ZnSO_4_, CuCl_2_, FeCl_3_, Ca(NO_3_)_2_, MgCl_2_, KCl, and NaCl were purchased from Maoming Chemical Reagent Factory (Tianjin, China). Tetracycline ointment (3%) was purchased from Guangdong Hengjian Pharmaceutical Co., Ltd. (Jiangmen, China) All chemicals are of AR grade, and no further purification is required.

### 2.2. Characterization

The morphology of the prepared materials was observed by the SU-8010 field emission scanning electron microscope (SEM, Hitachi, Tokoyo, Japan). The FT-IR spectra of 400 to 4000 cm^−1^ were recorded by a VERTEX70 FT-IR spectrophotometer (Bruker, Karlsruhe, Germany). The surface charge properties of raw materials in aqueous medium were measured by the Zetasizer ZS90 Nano (Malvern, Malvern, UK). U-3900 spectrophotometer (Hitachi, Japan) was used for UV–vis absorption spectrum. The fluorescence spectrum was measured by the RF-6000 fluorescence spectrophotometer (Shimadzu, Kyoto, Japan). XPS spectra were obtained by using an electronic spectrum of a Thermo Scientific K-Alpha spectrometer (Thermo Scientific, USA). The instrument measurement parameters were as follows: scan count: 10 times; spot size: 400 μm; pass energy: CAE 50.0; and step size: 0.10 eV. EXPEC 7000 (Hangzhou Convergence Technology, Hangzhou, China) is used for the quantification of ^152^Eu. Fluoromax-4 (Horiba, Kyoto, Japan) was used to measure the fluorescence lifetime. Tetracycline in actual samples was detected by L-2455 high-performance liquid chromatography (Hitachi, Tokoyo, Japan).

The parameters of HPLC are as follows: the chromatographic column is BioBasic-18 (4.6 mm × 250 mm, 5 μm), the mobile phase is oxalic acid solution (0.01 mol L^−1^)-acetonitrile-methanol (77:18:5), the injection volume is 20 μL, the scanning range is 300–400 nm, and the determination wavelength is 350 nm.

### 2.3. Preparation of BN-Eu

Using boric acid, urea, and europium chloride hexahydrate as raw materials, BN-Eu was synthesized by a one-step hydrothermal method. The specific steps were as follows: 200 mg of boric acid and 44.4 mg of urea were added to 10 mL of water, and the mixture was sonicated for 30 min. Then, 14.7 mg of europium chloride hexahydrate was stirred into the mixture to prepare a uniform and transparent solution. Pour the solution into 50 mL tetrafluoroethylene liner and react at 200 °C for 15 h. The cooled reactant was centrifuged at 6000 rpm for 10 min and washed with water three times. Finally, the obtained white solid was dried in a vacuum at 60 °C for 24 h. The synthesis procedure of BNQDs was identical to that for BN-Eu, except that EuCl_3_ was not added.

### 2.4. Ratiometric Fluorescence Detection of Tetracycline (TC)

First, 10 mg of BN-Eu was dispersed with 75 mL of deionized water to prepare a storage solution for later use. Next, 750 μL of storage solution was pipetted to ultrasonically disperse with 200 μL glycine–sodium hydroxide buffer solution (0.1 mol L^−1^, pH 9.5) for 10 min. Then, 50 μL of TC with different concentrations was added and reacted at room temperature for 5 min. The fluorescence data of 420–650 nm were collected by fluorescence spectrophotometer under the excitation wavelength of 390 nm.

### 2.5. Anti-Interference Performance of BN-Eu

To determine the anti-interference performance of BN-Eu, coexisting species, including metal ions, amino acid, and other antibiotics were added to the detection environment. The details of coexisting interferents include: 1 mmol L^−1^ of Cr^3+^, Co^2+^, Al^3+^, Cu^2+^, Zn^2+^, Cu^2+^, Fe^3+^, Ca^2+^, Mg^2+^, K^+^, Na^+^, glycine (Gly), leucine (Leu), histidine (His), phenylalanine (Phe), methionine (Met), glutathione (GSH), glucose (Glu), and fructose (Fru); 100 μmol L^−1^ of ampicillin (AMP), ciprofloxacin (Cip), enrofloxacin (Enr), sulfadiazine (SD), and vancomycin (Van); and 1 μmol L^−1^ of tetracycline substances, including doxycycline (DOX) and oxytetracycline (OXY).

### 2.6. Sample Treatment and Detection

A total of 6 mg tetracycline ointment (3%) was accurately weighed and ultrasonically dissolved into 30 mL diethyl ether for 15 min. Hydrochloric acid (0.1 mol L^−1^) was chosen as the extractant for the extraction of TC. The final obtained solution was detected by the above detection method, and the fluorescence emission spectrum of the system was recorded with the excitation wavelength of 390 nm. At the same time, the concentration of TC was quantified using high-performance liquid chromatography (HPLC) to verify the accuracy of the developed method.

### 2.7. Visual Detection for TC

First, 10 mg of BN-Eu was dispersed in 10 mL water to prepare the 1 mg mL^−1^ storage solution. Subsequently, 200 μL glycine–sodium hydroxide buffer solution (0.1 mol L^−1^, pH 9.5) was mixed with 750 μL storage solution, and then 50 μL TC with different concentrations were added into the mixed solution. The final TC concentrations in the sensing system were 0, 5, 10, 15, 20, 30, 40, and 50 μmol L^−1^, respectively. After reacting for 5 min at room temperature, a series of solutions with different concentrations were irradiated with a 365 nm hand-held ultraviolet lamp in the dark environment. Three groups of solution photos were taken in parallel by the smartphone. Just Color Picker was used to analyze the RGB values of photos, and investigate the relationship between TC concentration and R/B signal.

## 3. Results and Discussion

### 3.1. Characterization of BN-Eu

**Figure 1:**
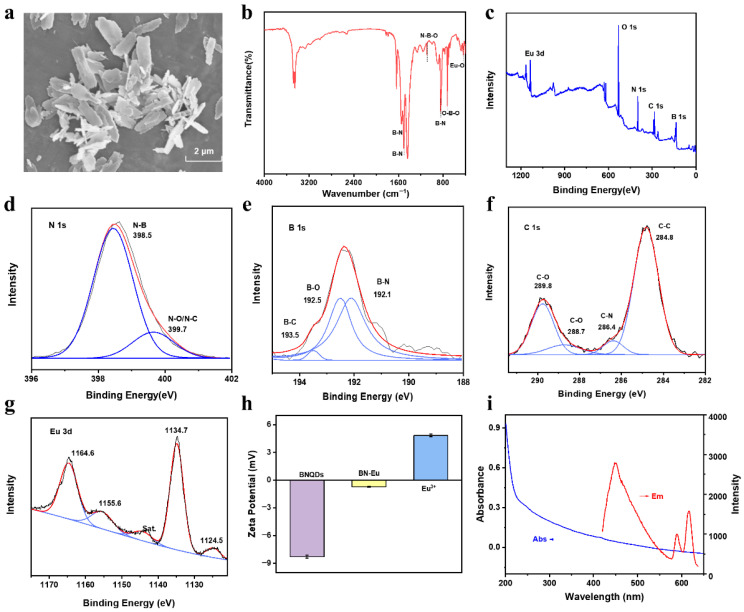
Characterization for BN-Eu: (**a**) SEM image of BN-Eu. (**b**) FT-IR spectrum of BN-Eu. (**c**) XPS survey spectrum of BN-Eu and the high-resolution spectra of (**d**) N 1s, (**e**) B 1s, (**f**) C 1s, and (**g**) Eu 3d. (**h**) Zeta potential of BNQDs, BN-Eu, and Eu^3+^. (**i**) UV−vis absorption (blue line) and fluorescence emission (red line) spectra of BN-Eu.

The BN-Eu materials were synthesized by utilizing a one-step hydrothermal method. Scanning electron microscope (SEM) images showed three-dimensional lamellar structure for BN-Eu at the micrometer level (Figure 1a). Fourier transform infrared (FT-IR) spectra are shown in Figure 1b to explore the chemical composition and functional groups of BN-Eu. There were tensile vibration absorption peaks (1549 cm^−1^ and 1437 cm^−1^) and bending vibration absorption peaks (843 cm^−1^) of B-N in the spectrum, and the characteristic absorption peaks at 1087 cm^−1^ and 725 cm^−1^ corresponding to N-B-O and O-B-O bonds, respectively, which indicated that there were abundant oxygen-containing functional groups and nitrogen-containing functional groups on the surface of boron nitride. The tensile vibration peak of Eu-O appeared at 434 cm^−1^, which proved that Eu^3+^ has been doped in boron nitride materials [21].

X-ray photoelectron spectrometer (XPS) spectrum of BN-Eu clearly indicates the presence of Eu, O, N, C, and B in BN-Eu (Figure 1c). The characteristic peaks of Eu 3d, O 1s, N 1s, C 1s, and B 1s appear at 1135.8, 531.9, 399.6, 284.8, and 192.6 eV, respectively. In addition, the high-resolution energy spectra of N 1s, C 1s, B 1s, and Eu 3d were analyzed in detail (Figure 1d–g). Among them, two distinct peaks of N 1s energy spectrum at 398.5 eV and 399.7 eV could be assigned to N-B and N-O/N-C bonds (Figure 1d). The peaks of B 1s energy spectrum at 192.1, 192.5, and 193.5 eV belonged to B-N, B-O, and B-C bonds, respectively (Figure 1e). The C 1s energy spectrum fitted four peaks at 284.8, 286.4, 288.7, and 289.8 eV, which were derived from C-C, C-N, and C-O bonds (Figure 1f). The binding energies at 1134.7 and 1164.6 eV were classified as Eu^3+^ 3d_5/2_ and Eu^3+^ 3d_3/2_, respectively. And the weak binding energy peaks at 1124.5 and 1155.6 eV were assigned to Eu^2+^ 3d_5/2_ and Eu^2+^ 3d_3/2_ (Figure 1c). These observations clearly indicated that europium mainly existed in the form of Eu^3+^ in BN-Eu [22,23,24]. To sum up, it was confirmed that Eu^3+^ had been doped in boron nitride by analyzing the elemental composition and chemical bonds in the XPS spectra [25].

Boron nitride with and without Eu^3+^ doping were synthesized under the same experimental conditions. As shown in Figure 1h, the Zeta potential of BNQDS and BN-Eu was analyzed. The Zeta potential of BNQDS was negative (−8.28 ± 0.15 mV). It increased due to the introduction of Eu^3+^, and the Zeta potential of BN-Eu was −0.72 ± 0.06 mV, which also demonstrated successful doping of positively charged Eu^3+^ [26]. In addition, the optical properties of BN-Eu were investigated by fluorescence spectra and UV–vis absorption spectra. As shown in Figure 1i, BN-Eu has no obvious characteristic absorption peak at 200–800 nm. The fluorescence signals of BN-Eu showed three fluorescence emission peaks at 449, 590, and 618 nm. The emission at 449 nm originated from the intrinsic emission of BN, and the emission at 590 nm and 618 nm originated from ^5^D_0_→^7^F_1_ and ^5^D_0_→^7^F_2_ transitions of Eu^3+^, respectively. The prepared BN-Eu probe exhibited excellent optical properties. And the fluorescence excitation wavelength was set at 390 nm for the ensuing studies.

### 3.2. Sensing Mechanism on Fluorescent Response of BN-Eu to TC

**Figure 2:**
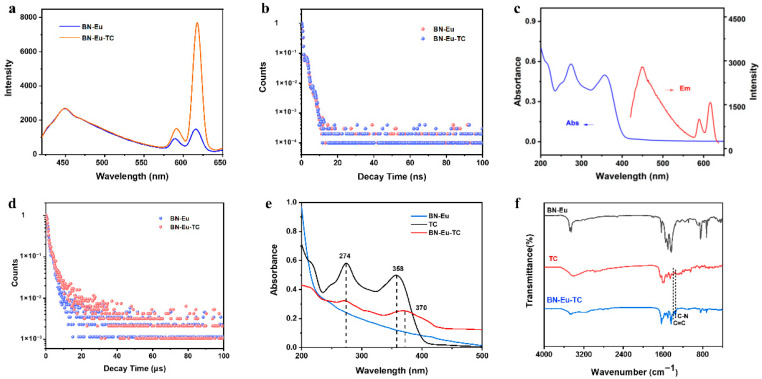
(**a**) Fluorescence emission spectra of BN-Eu and BN-Eu-TC. (**b**) Fluorescence lifetime decay curves of BN-Eu and BN-Eu-TC at 449 nm. (**c**) The fluorescence emission spectrum of BN-Eu (0.1 mg mL^−1^) and the UV–vis absorption spectrum of TC (1 μM, dispersed in water). (**d**) Fluorescence lifetime decay curves of BN-Eu and BN-Eu-TC at 618 nm. (**e**) UV–vis absorption spectra of BN-Eu, TC, and BN-Eu-TC. (**f**) FT-IR spectra of BN-Eu, TC, and BN-Eu-TC.

The feasibility of measuring TC by the BN-Eu probe was evaluated by measuring the fluorescence signal response of the BN-Eu solution before and after adding TC. As shown in Figure 2a, when tetracycline (TC) was introduced into the detection system based on BN-Eu, the fluorescence emission intensity (F_449_) of the detection system at 449 nm was almost unchanged, as the fluorescence emission intensity (F_618_) at 618 nm was significantly enhanced. Based on the above phenomenon, it was feasible to develop a TC ratiometric fluorescence sensing procedure using F_618_ as the response signal and F_449_ as the reference signal.

The detection mechanism of the BN-Eu sensing system was further explored. From the above results, it could be seen that there was no significant change in the fluorescence signal of the sensing system at 449 nm with the introduction of TC. As shown in Figure 2b, the fluorescence lifetimes of BN-Eu and BN-Eu-TC at 449 nm were 1.70 ns and 1.72 ns, respectively. In addition, there was no overlap between the fluorescence emission spectrum of BN-Eu and the UV–vis absorption spectrum of TC (Figure 2c). To sum up, there was no weak fluorescence quenching at 449 nm, which was consistent with the signal variation at 449 nm in the sensing process [27]. The enhanced fluorescence emission at 618 nm could be caused by the antenna effect. The fluorescence emission of BN-Eu at 590 nm and 618 nm was weak due to the coordinated water molecules. After introduction of TC, the β-diketonate configuration of TC could specifically chelate Eu^3+^ in BN-Eu and replace the water molecules [28,29], which made the characteristic energy transition from TC to Eu^3+^. The fluorescence sensitization triggered and promoted the enhancement of the characteristic fluorescence emission at 590 nm and 618 nm [30,31]. As depicted in Figure 2d, the fluorescence lifetime at 618 nm were significantly enhanced with introduction of TC (3.04 μs → 3.81 μs). In addition, the formation of BN-Eu-TC was also verified. By measuring the UV–vis absorption spectra of BN-Eu before and after adding TC (Figure 2e), it was found that BN-Eu had no obvious absorption peak, and TC had two characteristic absorption peaks at 274 nm and 358 nm. After adding TC, the characteristic absorption peak assigned to TC in the absorption spectrum of BN-Eu-TC shifted from 358 nm to 370 nm, indicating the formation of BN-Eu-TC [32]. As illustrated in Figure 2f, the FT-IR spectrum of BN-Eu-TC showed two new peaks at 1604 cm^−1^ and 1350 cm^−1^, which were ascribed to the stretching vibration of C=C and C-N in TC, further verifying the formation of BN-Eu-TC.

### 3.3. The Sensing Performance of TC Assay

**Figure 3:**
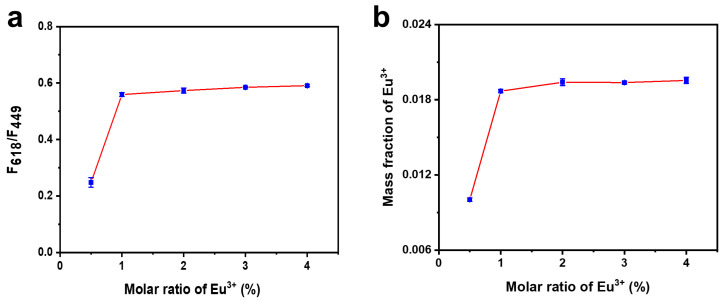
(**a**) Effect of Eu^3+^ doping molar ratio on the ratiometric fluorescence signal. (**b**) ^152^Eu content in BN-Eu detected by ICP-MS.

Probes are the key foundation for the construction of TC detection systems. To obtain the best performance of BN-Eu, the doping ratio of Eu^3+^ was optimized during the synthesis process of BN-Eu. As demonstrated in Figure 3a, with the increase in the molar ratio of Eu^3+^, the ratiometric fluorescence (F_618_/F_449_) gradually increased and finally reached a plateau. It might be because all the binding sites of boron nitride had been occupied by Eu^3+^, and the coordination of Eu^3+^ tended to be saturated. At the same time, the content of ^152^Eu in BN-Eu was also determined by inductively coupled plasma mass spectrometry (ICP-MS). As shown in Figure 3b, the mass fraction of Eu^3+^ in BN-Eu stayed basically unchanged, when the molar ratio of input was above 1%. It was consistent with the variation in ratiometric fluorescence signal. Therefore, 1% molar ratio of Eu^3+^ was selected as the optimal doping condition for preparing BN-Eu.

**Figure 4:**
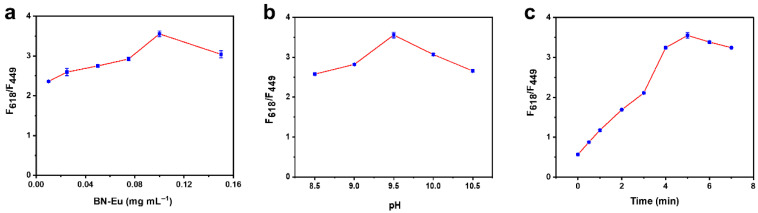
Effect of (**a**) BN-Eu concentration, (**b**) pH, and (**c**) reaction time on the ratiometric fluorescence signal (F_618_/F_449_) of TC sensing system.

The detection sensitivity for TC was affected by several experimental parameters, including concentration of probe, pH, and reaction time. In order to obtain the best detection performance of the sensing system for TC, the determination conditions were further scrutinized. The effect of probe concentration variation was investigated within 0.01–0.15 mg mL^−1^ (Figure 4a). It could be seen that a maximum signal response of ratiometric fluorescence was observed, when the concentration of BN-Eu was at 0.1 mg mL^−1^. The results clearly demonstrated 0.1 mg mL^−1^ as the optimal probe concentration for the assay. As illustrated in Figure 4b, the effect of pH values within a range of 8.5–10.5 was evaluated. It was seen that a maximum signal response was observed at pH 9.5. In addition, to evaluate the reaction time dependence of the detection system, the effect of reaction time on the ratiometric fluorescence response was investigated (Figure 4c). It could be observed that the fluorescence response signal of the detection system gradually increased with longer reaction time, reaching an optimal ratiometric fluorescence response at 5 min. It might be because when the reaction time reached 5 min, TC had fully bonded with BN-Eu to form new polymers BN-Eu-TC. Therefore, the reaction time of 5 min was chosen as the optimal time for TC detection.

**Figure 5:**
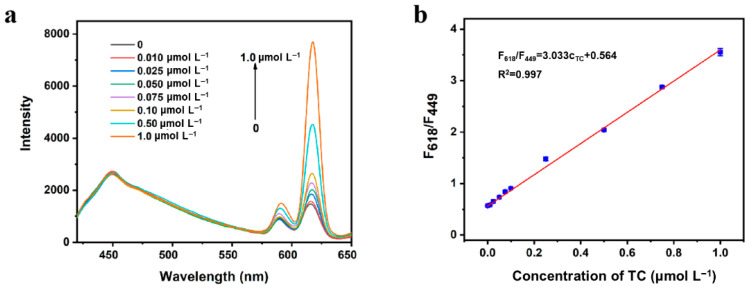
(**a**) The fluorescence spectra of the detection system with different concentrations of TC. (**b**) The linear relationship between ratiometric fluorescence signal (F_618_/F_449_) and TC concentration (0.010 to 1.0 μmol L^−1^).

Subsequently, under the optimal synthesis conditions and detection conditions as described above, the analytical performance of TC sensing was investigated (Figure 5a). The fluorescence intensity of the sensing system at 449 nm had no obvious change with the increase in TC concentration, while the fluorescence intensity at 618 nm increased significantly. The calibration curve method was applied for the TC determination. As shown in Figure 5b, there was a positive relationship between ratiometric fluorescence signal (F_618_/F_449_) and TC concentration. And a calibration plot was obtained within the range of TC (0.010 to 1.0 μmol L^−1^). The values were determined as follows: F_618_/F_449_ = 3.033 *C*_TC_ + 0.564 (R^2^ = 0.997) with a limit of detection (LOD) of 4.0 nmol L^−1^. Compared with the previously reported methods for TC detection (Table 1), it could be clearly seen that the ratiometric fluorescence detection method based on the antenna effect with BN-Eu as the probe had good sensitivity and a low detection limit. And the developed detection based on BN-Eu was among the hitherto reported sensitive strategies.

### 3.4. Selectivity Assay of TC by the BN-Eu Sensing Platform

**Figure 6:**
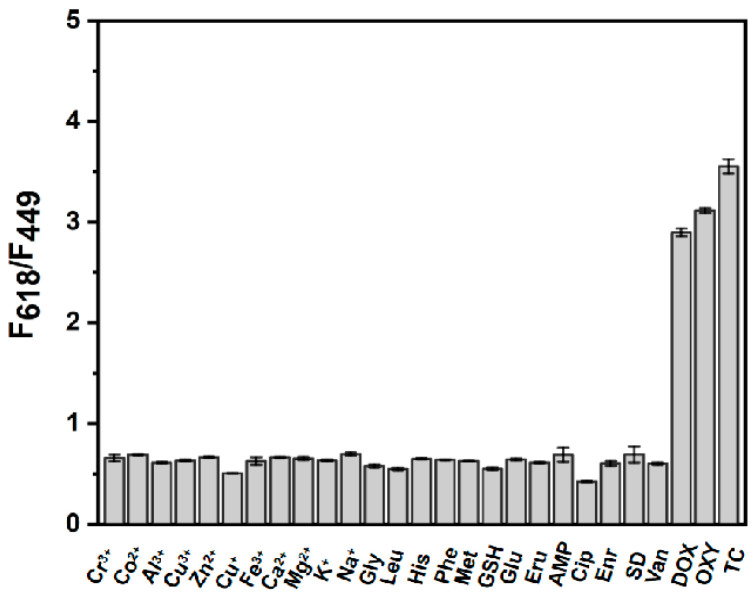
The signal response of the ratiometric fluorescent probe based on BN-Eu to tetracyclines and other interfering substances. Interference substance: Cr^3+^, Co^2+^, Al^3+^, Cu^2+^, Zn^2+^, Cu^+^, Fe^3+^, Ca^2+^, Mg^2+^, K^+^, Na^+^, Gly, Leu, His, Phe, Met, GSH, Glu, and Fru (1 mmol L^−1^); AMP, Cip, Enr, SD, and Van (100 μmol L^−1^); Tetracycline antibiotics: DOX, OXY, and TC (1 μmol L^−1^).

For the purpose of detecting TC in actual samples, favorable anti-interference capability of the sensing system is extremely important. In the present case, the effect of some frequently encountered species in sample matrixes, e.g., cationic species, amino acids, as well as small molecules were investigated as illustrated in Figure 6. The results clearly indicated that the presence of the above species at certain concentration levels had a negligible effect on the ratiometric fluorescence signal of BN-Eu except tetracycline antibiotics. The favorable selectivity and anti-interference capability of the assay system ensured its application in the detection of TC in actual samples. The inability to distinguish specific tetracyclines is a limitation in the developed analytical method based on BN-Eu. Our group is currently dedicated to developing fluorescent sensor arrays to further distinguish tetracyclines.

### 3.5. Assay of TC in Actual Samples

**Figure 7:**
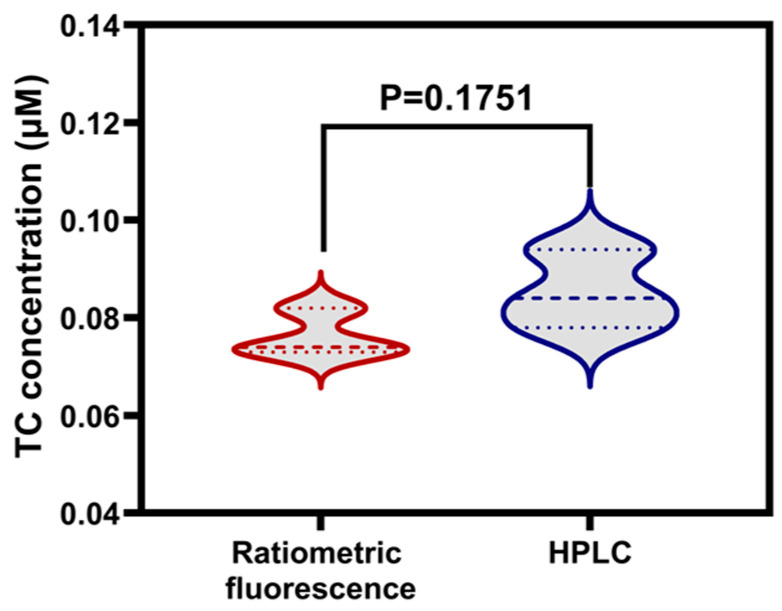
The violin plot of the detection methods (ratiometric fluorescence method and HPLC).

The practical applicability and reliability of the TC detection system based on BN-Eu was evaluated through determination of TC in tetracycline ointment, and the recovery was investigated using the standard solutions of tetracycline ointment spiked with TC at three levels (0.25, 0.50, and 0.75 μmol L^−1^). As shown in Table 2, the recovery rate of TC in tetracycline ointment was 89.9% to 99.8%, and the relative standard deviation (RSD) was less than 5.9% (*n* = 3). The concentration of the original sample calculated from the spiked recovery experiment (e.g., 0.071 ± 0.020 μM and 0.075 ± 0.025 μM) exhibited significantly increased uncertainty compared to the direct measurement (0.076 ± 0.005 μM). This phenomenon could be caused by matrix effects. In addition, the results obtained from the two detection methods were analyzed by the statistical t-test. Figure 7 showed that there is no significant difference between the two methods. Therefore, the sensing system can accurately measure TC content, and it is feasible to be applied to the determination of TC content in actual samples.

### 3.6. Visual Detection of TC Based on Smartphone Analysis Device

**Figure 8:**
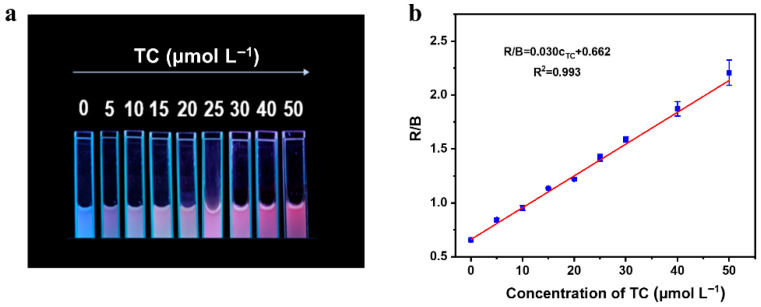
Under the irradiation of 365 nm light, the smartphone took photos of the solution based on BN-Eu sensing TC and established the relationship between TC concentration and RGB value of photos. (**a**) The corresponding photos of the detection system taken by the smartphone for different concentrations of TC concentration. (**b**) The relationship between the R/B value of the sensing system and the TC concentration.

Recently, visualization-based sensitive sensors have represented a major research direction in portable detection. As the TC concentration increased, the reaction system underwent a gradual color change from blue to red under the 365 nm irradiation (Figure 8a). The color change could be monitored in real-time by a smartphone camera. After taking photos by smartphone, the RGB values were analyzed by Just Color Picker software. The value of R/B was chosen as the response signal to investigate its relationship with TC concentration. It was found that TC concentration had a good linear relationship with an R/B value in the range of 0–50 μmol L^−1^, the linear regression equation was R/B = 0.030 c_TC_ + 0.662 (R^2^ = 0.993) and LOD was 1.4 μmol L^−1^ (Figure 8b). Hence, the portable determination of TC concentration was realized according to the color change in the solution and software analysis.

## 4. Conclusions

BN-Eu was synthesized by a simple hydrothermal method in this work. Based on the antenna effect between BN-Eu and TC, a ratiometric fluorescence sensor was successfully constructed for TC detection. This facile sensing mechanism effectively provided a selective and sensitive assay protocol for the TC detection. This work provided new perspectives for future development of advanced fluorescence sensors for the detection of antibiotic residue. In addition, based on the color change in the process of TC detection, the on-site and real-time TC visual detection was also realized by the smartphone.

## Figures and Tables

**Table 1:** Performance comparison between BN-Eu and other previously reported materials related to tetracycline fluorescence detection.

Materials	Mode	Detection	Linear Range (μM)	LOD (nM)	Ref.
BNQDs-Eu^3+^	ratiometric	tetracycline	0–50	19	[23]
Luminol-Eu-CitNa	ratiometric	tetracycline	0.5–80	39	[25]
ZnO/Eu	ratiometric	tetracycline	0.005–3	4	[21]
g-C_3_N_4_/Eu^3+^	ratiometric	tetracycline	0.25–80	6.5	[16]
CDs-CuInS_2_/ZnS	off-on	chlortetracycline	1–50	36	[13]
N-CDs	ratiometric	tetracycline	0.05–324	22.7	[11]
Ir(III)@SiNPs-Eu^3+^	ratiometric	tetracycline	0.01–20	4.9	[17]
BN-Eu	ratiometric	tetracycline	0.01–1.00	4.0	This work

**Table 2:** Determination of TC in tetracycline ointment.

Detection Method	Spiked (μM)	TC Concentration (μM)	Recovery (%)	RSD (%)
Ratiometric fluorescence	—	0.076 ± 0.005	—	—
	0.25	0.321 ± 0.019	98.0	5.9
	0.50	0.575 ± 0.024	99.8	4.2
	0.75	0.750 ± 0.016	89.9	2.1
HPLC	—	0.085 ± 0.008	—	—
	0.25	0.338 ± 0.014	101.2	4.1
	0.50	0.608 ± 0.020	104.6	3.3
	0.75	0.828 ± 0.027	99.1	3.3

## Data Availability

The data that support the findings of this study are available from the corresponding author upon reasonable request.
